# The PAS domains of the major sporulation kinase in *Bacillus subtilis* play a role in tetramer formation that is essential for the autokinase activity

**DOI:** 10.1002/mbo3.481

**Published:** 2017-04-27

**Authors:** Brittany Kiehler, Lindsey Haggett, Masaya Fujita

**Affiliations:** ^1^ Department of Biology and Biochemistry University of Houston Houston TX USA

**Keywords:** *Bacillus subtilis*, histidine kinase, response regulator, signal transduction, sporulation

## Abstract

Sporulation in *Bacillus subtilis* is induced upon starvation. In a widely accepted model, an N‐terminal “sensor” domain of the major sporulation kinase KinA recognizes a hypothetical starvation signal(s) and autophosphorylates a histidine residue to activate the master regulator Spo0A via a multicomponent phosphorelay. However, to date no confirmed signal has been found. Here, we demonstrated that PAS‐A, the most N‐terminal of the three PAS domains (PAS‐ABC), is dispensable for the activity, contrary to a previous report. Our data indicated that the autokinase activity is dependent on the formation of a functional tetramer, which is mediated by, at least, PAS‐B and PAS‐C. Additionally, we ruled out the previously proposed notion that NAD ^+^/NADH ratio controls KinA activity through the PAS‐A domain by demonstrating that the cofactors show no effects on the kinase activity in vitro. In support of these data, we found that the cofactors exist in approximately 1000‐fold excess of KinA in the cell and the cofactors’ ratio does not change significantly during growth and sporulation, suggesting that changes in the cofactor ratio might not play a role in controlling KinA activity. These data may refute the widely‐held belief that the activity of KinA is regulated in response to an unknown starvation signal(s).

## 1 Introduction

When the Gram‐positive soil bacterium *Bacillus subtilis* is exposed to starvation conditions, the cell switches from medial to asymmetric division to produce a larger mother cell and a smaller forespore, leading to the production of a mature spore (Piggot & Hilbert, 2004). In the early stages of nutrient limitation, sporulation is activated by a multicomponent phosphorelay, a complex version of bacterial two‐component signal transduction system composed of the major sporulation kinase KinA, two intermediate phosphotransferases (Spo0F and Spo0B), and a response regulator (Spo0A) (Burbulys, Trach, & Hoch, 1991; Hoch, 1993). KinA is a cytosolic histidine kinase (Jiang, Shao, Perego, & Hoch, 2000), in which a histidine residue at the C‐terminal DHp (dimerization/histidine phosphorylation) domain is autophosphorylated with ATP in a trans manner (Devi, Kiehler, Haggett, & Fujita, 2015) and then transfers the phosphoryl group to the downstream components (Spo0F, Spo0B, and Spo0A) in a sequential His‐Asp‐His‐Asp phosphorelay, resulting in the activation of Spo0A as a phosphorylated form (Spo0A~P) (Burbulys et al., 1991). It is widely believed that the autophosphorylation activity of KinA is triggered when its N‐terminal “sensor” domain, which contains three PAS domains (PAS‐ABC), receives an as yet unknown starvation signal(s), analogous to other bacterial two‐component systems (Stock, Robinson, & Goudreau, 2000). However, the detailed mechanisms of the kinase activation, including how the putative “sensor” domain recognizes the starvation signal(s) and how the autokinase activity is then triggered, remain elusive because the molecular identity of the starvation signal(s) is as yet unclear.

To date, multiple pieces of evidence have suggested the importance of Spo0A activation in regulating the initiation of sporulation upon starvation. First, the cellular level of Spo0A~P increases gradually over the course of starvation in the cell (Fujita & Losick, 2005). Second, when the cellular concentration of Spo0A~P reaches a certain level (threshold), the position of the cell division site switches from medial to polar, resulting in the arrest of vegetative growth (Fujita, Gonzalez‐Pastor, & Losick, 2005). Third, after asymmetric cell division, the level of Spo0A~P continues to increase only in the mother cell (larger compartment), while Spo0A disappears in the forespore (smaller compartment) (Fujita & Losick, 2003). Fourth, this spatiotemporal increase in Spo0A activity is essential for proper cell‐fate determination (Kovacs, 2016; Vishnoi et al., 2013).

While the starvation signal(s) is unknown, recent studies have attempted to address the mechanisms of KinA activation. Some key findings are that, first, there is no significant difference in the level of kinA transcripts between growing and sporulating cells (Eswaramoorthy, Duan et al., 2010). Second, both the protein level and activity of KinA increase slightly and gradually over the course of nutrient starvation (Eswaramoorthy, Dinh, Duan, Igoshin, & Fujita, 2010; Eswaramoorthy, Duan et al., 2010). Third, a certain threshold level of KinA protein is necessary and sufficient to trigger phosphorelay signaling and sporulation (Eswaramoorthy, Duan et al., 2010; Narula et al., 2016). And fourth, the N‐terminal domain of KinA plays a role in the formation of a stable tetramer complex, but may not contribute a signal sensing function to activate the enzyme's C‐terminal catalytic domain (Eswaramoorthy & Fujita, 2010; Eswaramoorthy, Guo, & Fujita, 2009; Eswaramoorthy et al., 2011). Thus, based on these observations, the threshold level of KinA appears to be a primary regulator to produce Spo0A~P beyond a critical level to direct the expression of the Spo0A‐controlled sporulation genes.

Prior in vivo evidence suggests that the most N‐terminal PAS domain of KinA, PAS‐A, is dispensable for the enzyme's autokinase activity as well as for sporulation (Eswaramoorthy & Fujita, 2010; Eswaramoorthy et al., 2009). These results are in agreement with an in vitro study reported by Winnen, Anderson, Cole, King, & Rowland (2013). In contrast, several of the previous studies have indicated that PAS‐A is essential for the autokinase activity (Kolodkin‐Gal et al., 2013; Lee et al., 2008; Stephenson & Hoch, 2001; Wang et al., 2001). Among them, Lee et al., (2008) reported that the purified PAS‐A domain forms a dimer by itself. However, studies by the Hoch group indicate that PAS‐A is monomeric and possesses ATP‐binding and nucleoside diphosphate kinase‐like activities (Stephenson & Hoch, 2001). These two groups also demonstrated that the PAS‐A domain is essential for the autokinase activity (Lee et al., 2008; Stephenson & Hoch, 2001; Wang et al., 2001). In support of their findings, a more recent paper by Kolodkin‐Gal et al., (2013) reported that the removal of PAS‐A from KinA impairs sporulation, although the key data were not shown. Furthermore, they proposed that KinA activity is inhibited by high NAD^+^/NADH ratio through the PAS‐A domain (Kolodkin‐Gal et al., 2013). It is noted that the PAS‐A deletion construct (aa 151–606) used in the study by Lee et al. (2008) featured an extra 8aa deletion which extended into the PAS‐B domain (aa 143–150), giving it a structure more similar to our autokinase‐deficient PAS‐AB mutant (aa 259–606) (Eswaramoorthy et al., 2009). These facts suggest that the Lee et al. strain may not be truly representative of a PAS‐A deletion mutant. Multiple distinct models have been proposed to describe potential roles for the PAS‐A domain by individual research group as a result of the above discrepancies.

Nevertheless, based mainly on our in vitro and in vivo data (Eswaramoorthy & Fujita, 2010; Eswaramoorthy, Dinh, et al., 2010; Eswaramoorthy et al., 2009; Eswaramoorthy et al., 2011; Eswaramoorthy, Duan et al., 2010; Narula et al., 2016), we have proposed a model in which a threshold level of the major kinase KinA acts as a molecular switch to determine entry into sporulation, which may rule out the possibility that KinA is activated by sensing an as yet unidentified starvation signal(s) with the N‐terminal PAS domains. Our model is supported by more recent data, in which cell growth slows down upon starvation, leading to a relative increase in the cellular concentration of KinA as its production rate remains constant (Narula et al., 2016). Thus, when the KinA threshold is reached, the flow of phosphoryl groups through the phosphorelay proceeds toward the production of the phosphorylated Spo0A sufficient to activate transcription of the high‐threshold genes required for sporulation.

In this paper, to provide direct supporting evidence for our proposed model, we further explored the role of the N‐terminal PAS domains of KinA in its enzymatic activity. We first investigated whether the PAS‐A domain is dispensable for the autokinase activity using an in vitro assay. Second, we tested whether KinA activity is dependent on the tetramer formation mediated by the N‐terminal PAS domains in vivo. Finally, with in vivo and in vitro biochemical characterizations, we attempted to rule out the previously proposed possibility, in which KinA activity is controlled by NAD^+^/NADH ratio through the PAS‐A domain (Kolodkin‐Gal et al., 2013).

## 2 Experimental procedures

### 2.1 Strains, plasmids, and oligonucleotides

All *B. subtilis* strains were derived from the prototrophic strain PY79 (Youngman, Perkins, & Losick, 1984). Details of the constructions are available upon request. All plasmid constructions were performed in *Escherichia coli* DH5α using standard methods. The *E. coli* BL21(DE3) pET vector system (Novagen) was used for protein overexpression. The strains and plasmids used in this study are listed in Tables S1 and S2, respectively. Oligonucleotides used for plasmid construction are listed in Table S3.

### 2.2 Media, culture conditions, protein expression, and protein purification

To induce protein synthesis in *B. subtilis* cells, a gene of interest was placed under the control of an isopropyl‐β‐D‐thiogalactopyranoside (IPTG)‐inducible hyper‐spank promoter (Phy‐spank) in a plasmid that integrates stably into the *amyE* locus of the chromosomal DNA. After plasmid integration into the host genome, the desired concentration of IPTG was added to Luria‐Bertani (LB) cultures during the exponential growth phase (optical density at 600 nm [OD600], 0.5) at 37°C as the rich medium conditions. To induce protein synthesis in *E. coli*, the *E. coli* BL21(DE3) pET vector system was used according to manufacturer's protocol (Novagen). For his‐tagged protein purification, the cells were harvested after 2 hr of induction and the induced proteins were purified by Ni‐NTA beads according to the manufacturer's protocol (Invitrogen). For NAD^+^/NADH assay, LB and DSM (Difco‐sporulation medium, also known as Schaeffer's sporulation medium) (Cutting & Vander Horn, 1990) were used for nutrient‐rich and sporulation conditions. For the reverse phosphotransferase reaction assay, sporulation was induced by the resuspension method (Sterlini & Mandelstam, 1969).

### 2.3 Immunoblot analysis

Whole‐cell lysates for immunoblot analysis were prepared by sonication. Protein concentration was measured by the Bradford method (Pierce). Total proteins were subjected to SDS‐PAGE and transferred to a nitrocellulose filter. Immunoblot analysis was done with polyclonal anti‐GFP antibodies (a gift from David Rudner). Alkaline phosphatase‐coupled secondary antibodies (Anti‐Rabbit IgG, Promega) were used for recognizing the primary antibodies and detected using substrate nitro blue tetrazolium–5‐bromo‐4‐chloro‐3‐indolyl phosphate (NBT‐BCIP) (Promega).

### 2.4 Protein cross‐linking

Protein cross‐linking with bis‐maleimidohexane (BMH; Pierce) was performed as described previously (Eswaramoorthy et al., 2009). A regression line of the log molecular mass versus the relative migration distance was generated using the HiMark Protein Standard (Thermo Fisher Scientific) and monomer form of KinA‐GFP, KinA^ΔPAS‐A^‐GFP, KinA^ΔPAS‐AB^‐GFP, KinA^ΔPAS‐ABC^‐GFP, and KinA_N_‐GFP. Strains MF3352 (KinA‐GFP), MF3353 (KinA^ΔPAS‐A^‐GFP), MF3356 (KinA^ΔPAS‐AB^‐GFP), MF3359 (KinA_C_‐GFP), and MF3360 (KinA_N_‐GFP) were used.

### 2.5 Blue native polyacrylamide gel electrophoresis

The blue native PAGE (BN‐PAGE) assay was performed according to the protocol provided by Novex (Thermo Fisher Scientific). In brief, the C‐terminal hexahistidine‐tagged GFP fusion proteins of KinA_N_‐GFP‐his6 (MF7622), PAS‐BC‐GFP‐his6 (MF7623), PAS‐C‐GFP‐his6 (MF7624), and KinA_C_‐GFP‐his6 (i.e., ΔPAS‐ABC, MF7625) were expressed under the control of an IPTG‐inducible Phy‐spank promoter in *B. subtilis*. To avoid the possible PAS domains‐mediated heterocomplex formation between the native KinA (i.e., non‐his tagged) and the his‐tagged protein, a gene for the native KinA was deleted (*ΔkinA::tet*). The proteins examined were purified using Ni‐NTA beads (Promega). The sample eluates were subjected to a 4–16% gradient BN‐PAGE gel (Novex, Thermo Fisher Scientific), followed by immunoblot analysis with polyclonal anti‐GFP antibodies (Fujita & Losick, 2002). A regression line of the log molecular mass versus the relative migration distance was generated using the NativeMark Unstained Protein Standard (Thermo Fisher Scientific).

### 2.6 In vivo assay of reverse phosphotransfer reaction

Strains MF3386 (no IPTG‐inducible system), MF3388 (KinA_N_), MF3387 (KinA_C_), MF7679 (KinA), MF7680 (KinA^ΔPAS‐A^), and MF7681 (KinA^ΔPAS‐AB^), containing the kinA‐null mutation (*ΔkinA*) and PspoIIQ‐gfp, were used for the reverse phosphotransfer assay. Each of the genes for the tested proteins was placed under the control of an IPTG‐inducible Phy‐spank promoter in *B. subtilis*. Overexpression of the protein was induced by addition of IPTG at a final concentration of 0.5 mmol L^−1^ after sporulation by resuspension (Sterlini & Mandelstam, 1969). Sporulation efficiency was measured by counting cells expressing GFP signals under the control of the forespore‐specific spoIIQ promoter (PspoIIQ).

### 2.7 In vitro phosphorylation

The C‐terminal hexahistidine‐tagged KinA and KinA^ΔPAS‐A^ proteins were expressed under the control of an IPTG‐inducible Phy‐spank promoter in *B. subtilis*. To avoid the possible PAS domains‐mediated heterocomplex formation between the native KinA (i.e., non‐his‐tagged) and the his‐tagged protein, a gene for the native KinA was deleted (*ΔkinA::tet*). Spo0F, Spo0B, Spo0A, and KinC^ΔTM−1+2^ were purified from the *E. coli* overexpression systems (Table S1) as reported (Devi, Vishnoi, Kiehler, Haggett, & Fujita, 2015; Fujita & Losick, 2003). The phosphorylation assays were performed in a 20 μl final reaction volume containing 50 mmol L^−1^ Tris‐HCI (pH 8.0), 5 mmol L^−1^MgCI_2_, 10% glycerol. Proteins used for the phosphorylation assays were 0.2 μmol L^−1^ KinA, 0.2 μmol L^−1^ KinA^ΔPAS‐A^, 0.2 μmol L^−1^ KinC^ΔTM−1+2^, 0.2 μmol L^−1^ Spo0F, 0.2 μmol L^−1^ Spo0B, and 2 μmol L^−1^ Spo0A. The reaction was initiated by the addition of ATP to a final concentration of 0.4 mmol L^−1^ containing 1 μCi of [γ‐^32^P] ATP (purchased from Perkin‐Elmer, 3000 Ci/mmol, 10 mCi/ml). Reaction mixtures were incubated for 30 min at 30°C and then stopped by adding SDS‐loading buffer (Laemmli, 1970). Samples were subjected to electrophoresis through an 18% SDS–polyacrylamide gel, and radioactive proteins were visualized by autoradiography.

### 2.8 Determination of cellular NAD^+^ and NADH concentrations

Cellular NAD^+^ and NADH concentrations were determined in *B. subtilis* PY79 using the NAD/NADH Quantification Kit provided by Sigma‐Aldrich (Catalog Number MAK037, St Louis, MO). In brief, growing vegetative cells were cultured in LB for 4 hr at 37°C and sporulating cells were prepared in DSM for 5 hr (2 hr after onset of sporulation) at 37°C. Cell numbers were determined as colony‐forming unit (CFU) with serial dilution and plate counts. One aliquot of cells (approximately 1 × 10^7^ cells) was lysed with two freeze‐thaw cycles in the NAD/NADH extraction buffer, and then total extracts were filtered through 3‐kD cutoff filters (Amicon Ultra‐5, Sigma‐Aldrich) to recover only the unbound free forms of NAD^+^ and NADH. Half of the flow‐through lysate was used to determine total NAD concentrations (NAD^+^ and NADH). The other half was heated to 60°C for 5 min and used to determine NADH concentrations. NADH standards were prepared as 0 (blank), 20, 40, 60, 80, and 100 pmole/reaction. A reaction mixture containing cycling enzyme provided by the kit was added to each sample and allowed to convert NAD^+^ to NADH at 25°C for 5 min. NADH developer supplied in the kit was then added to the sample reactions and the NADH standards were incubated for 15 min at 25°C and read at 450 nm. Finally, the NAD^+^ concentration per reaction was determined by subtracting NADH concentration per reaction from total NAD (NAD^+^ and NADH) concentration per reaction. Intracellular concentrations were calculated by taking into account the number of cells per reaction (determined with CFU) and cell volume (1 × 10^‐15^ L). *E. coli* DH5α was used as a control (Bennett et al., 2009).

## 3 Results

### 3.1 The most amino‐terminal PAS‐A domain is dispensable for autokinase activity of KinA

**Figure 1:**
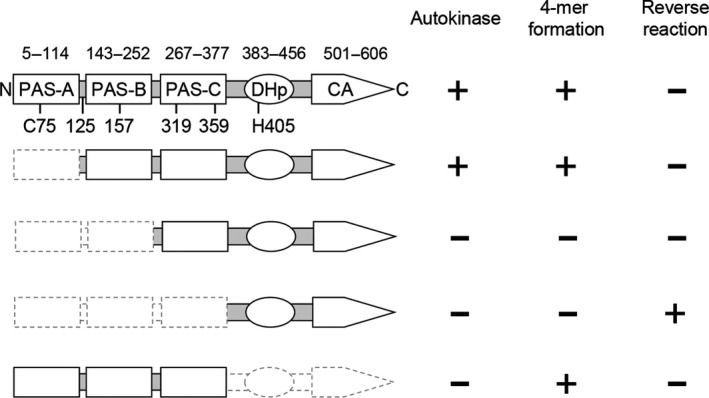
Summary diagram of KinA and its truncated mutants. The N‐terminal domain is subdivided into three PAS domains (PAS‐ABC). The C‐terminal domain is subdivided into DHp (dimerization and histidine phosphotransfer) domain and CA (catalytic ATP‐binding) domain (Eswaramoorthy et al., 2009). Domain boundaries indicate the amino acid residue numbers from N‐terminus. Positions of cross‐linkable cysteine residues with bis‐maleimidohexane (BMH) are indicated in the N‐terminal domain. The histidine phosphorylation site is located at His405 as indicated. As a summary of this study, autokinase activity, tetramer (4‐mer) formation, and reverse phosphotransfer activity (reverse reaction from Spo0F~P to KinA_C_) are scored with a plus sign or a minus sign to denote detectable or not, respectively

**Figure 2:**
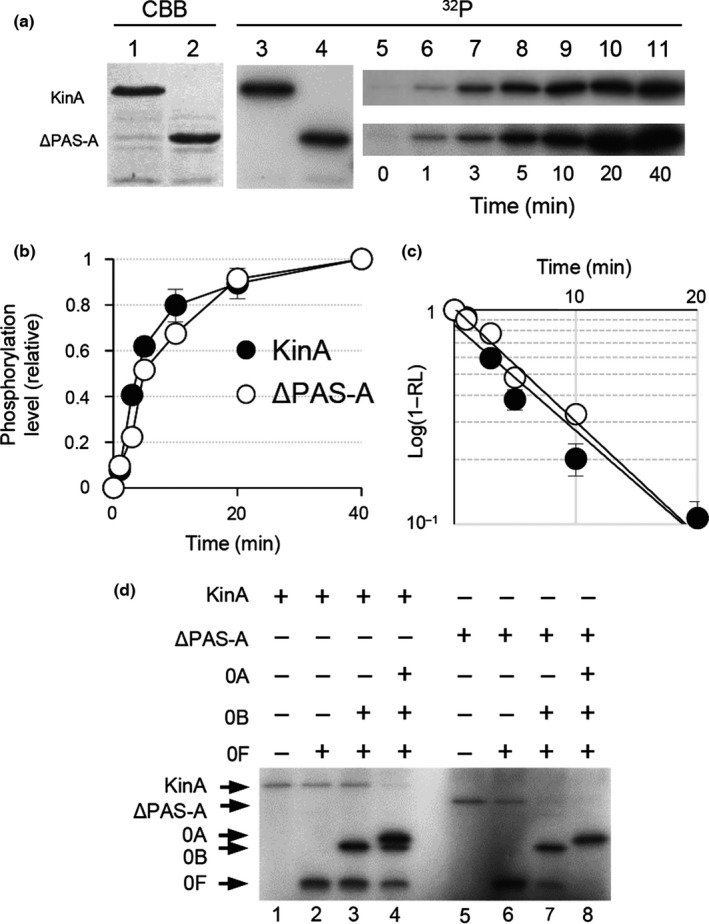
In vitro autophosphorylation assay for PAS‐A domain deletion form of KinA (KinA^Δ^ ^PAS^ ^‐A^). (a) Autophosphorylation activities of KinA and KinA^Δ^ ^PAS^ ^‐A^ (ΔPAS‐A) were measured in an in vitro reaction. Each of the purified proteins was analyzed by SDS‐PAGE (lanes 1 and 2, 0.1 μg protein stained with Coomassie brilliant blue, CBB in panel a) and with in vitro autophosphorylation in the presence of [γ‐^32^P]ATP followed by autoradiography of SDS‐PAGE (lanes 3 and 4, ^32^P in panel a). For kinetic analysis, at the indicated times, aliquots were removed from the in vitro autophosphorylation reaction mixture, mixed with SDS‐PAGE sample buffer to stop the reaction, and analyzed by SDS‐PAGE followed by autoradiography (lanes 5–11). (b) Relative phosphorylation levels over time of reaction. The fractions of phosphorylation levels plotted on the y‐axis in the graph were defined as the ratio of each of the radiolabeled proteins at the indicated time point to the maximum level of that present at the end point of the reaction and are expressed as the relative level (RL). (c) Graph indicates the semilogarithmic plot of the value of (1‐RL) as a function of time. The estimated values of *k*obs (pseudo‐first‐order rate constant) for the autophosphorylation of KinA and KinA^Δ^ ^PAS^ ^‐A^ were calculated from the slopes. The symbols are the same as in (b). (d) Spo0A phosphorylation by KinA and KinA^Δ^ ^PAS^ ^‐A^ through phosphorelay. The purified proteins as indicated on the top were incubated with [γ‐^32^P]ATP as described in Materials and Methods. (+) and (−) indicate with and without proteins, respectively. Whole reaction mixtures were analyzed by SDS‐PAGE followed by autoradiography. Each of the gel images displayed is one of the representative results (a and d). The mean from three independent experiments with standard error is shown (b and c)

To conclude the controversial debate on whether the PAS‐A domain is required for the autokinase activity (Eswaramoorthy et al., 2009; Kolodkin‐Gal et al., 2013; Lee et al., 2008; Stephenson & Hoch, 2001; Wang et al., 2001; Winnen et al., 2013), we first performed quantitative in vitro biochemical characterizations of KinA. For this, we constructed a series of *B. subtilis* strains expressing his‐tagged phosphorelay proteins under the control of an IPTG‐inducible promoter (Figure 1). Major advantages of this method are that: (1) native proteins can be easily purified from *B. subtilis* cells under physiological conditions and (2) by changing the culture conditions and sampling times, proteins can be purified at different physiological states (e.g., under growth, sporulation, aerobic, or anaerobic conditions). *B. subtilis* cells harboring the IPTG‐inducible histidine‐tagged KinA and KinA^ΔPAS‐A^ were cultured in a nutrient‐rich LB medium to exclude any possible involvement of unknown starvation signals. IPTG was added to a mid‐logarithmic phase culture at a concentration of 0.5 mmol L^−1^ to induce proteins and collected 2 hr after the addition of IPTG. His‐tagged KinA and KinA^ΔPAS‐A^ were purified from the cell extracts using Ni‐NTA beads (Figure 2a, lanes 1 and 2). To determine whether KinA^ΔPAS‐A^ is active similarly in the wild‐type KinA, we performed an in vitro autophosphorylation assay under standard conditions (Fujita & Losick, 2003). We found that the purified KinA and KinA^ΔPAS‐A^ were both phosphorylated in the presence of radiolabeled ATP, in agreement with a previous report by Winnen et al., (2013) (Figure 2a, lanes 3 and 4). To further characterize the detailed kinetics of the purified KinA and KinA^ΔPAS‐A^, we examined the time course of phosphorylation of the two proteins in response to ATP (Figure 2a, lanes 5‐11). We found that the phosphorylation levels of KinA^ΔPAS‐A^ over the course of reactions were similar to those observed for KinA (Figure 2b). Based on these results, the estimated values of *k*obs (pseudo‐first‐order rate constant) for the autophosphorylation of KinA and KinA^ΔPAS‐A^ were determined to be indistinguishable from each other (0.12 min^−1^) (Figure 2c). Finally, we tested whether Spo0A can be phosphorylated by KinA^ΔPAS‐A^ through the action of the phosphorelay. As shown in Figure 2d, we found that Spo0A was phosphorylated in the presence of KinA^ΔPAS‐A^, similar to wild‐type KinA. Taken together, these results indicated that the purified KinA^ΔPAS‐A^ displayed similar activity and kinetics to those of the wild‐type enzyme, indicating that the PAS‐A domain is dispensable for the autokinase activity in an in vitro assay, as it was demonstrated to be in an in vivo assay (Eswaramoorthy & Fujita, 2010; Eswaramoorthy et al., 2009, 2011).

### 3.2 KinA^ΔPAS‐A^ is able to form a homotetramer as a functional unit, similar to wild‐type KinA

**Figure 3:**
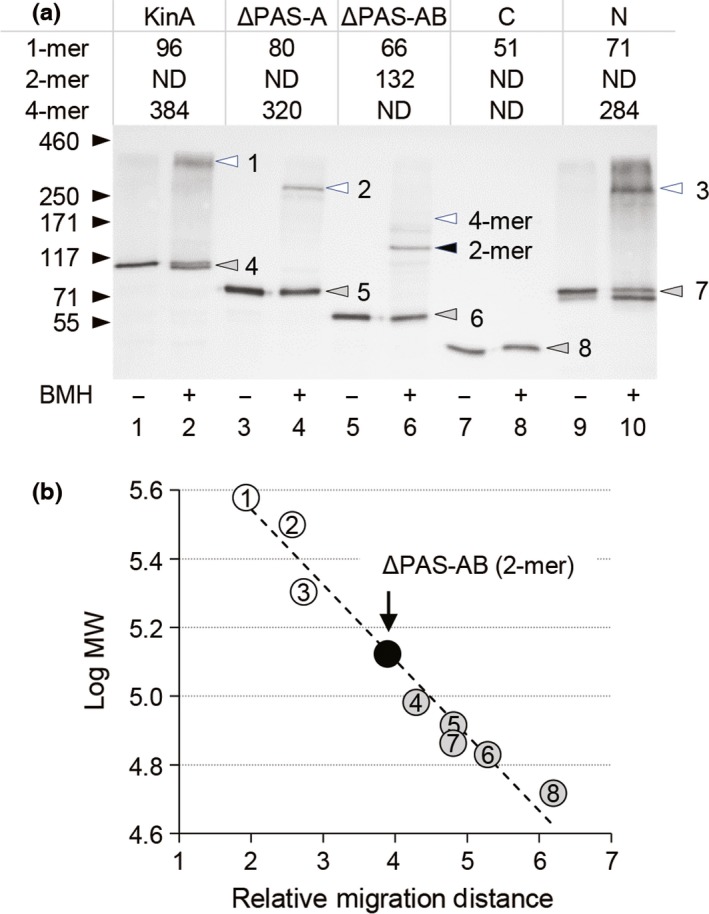
Complex formation of KinA and its truncated proteins analyzed by cross‐linking. (a) Cell extracts were prepared from strains MF3352 (full‐length KinA‐GFP) (lanes 1 and 2), MF3353 (KinA^Δ^ ^PAS^ ^‐A^‐GFP) (lanes 3 and 4), MF3356 (KinA^Δ^ ^PAS^ ^‐^ ^AB^‐GFP) (lanes 5 and 6), MF3359 (KinA^Δ^ ^PAS^ ^‐^ ^ABC^‐GFP, KinA_C_‐GFP) (lanes 7 and 8), and MF3360 (KinA_N_‐GFP) (lanes 9 and 10) were grown in LB medium in the presence of IPTG (10 μmol L^−1^). The extracts were then processed for the BMH cross‐linking reaction as described in Materials and Methods. Samples were separated by SDS‐PAGE (10%), followed by immunoblot analysis with anti‐GFP antibodies. ‐, no BMH; +, with BMH. The molecular mass of each detected protein is indicated as kDa in the table on the top. Protein bands corresponding to monomer (gray), dimer (black), and tetramer (white) are indicated by arrowheads on the gel image. In lane 6, the position corresponding to the tetramer size is indicated, while the actual band was not detected. The calculated molecular masses are good agreement with the molecular mass standards indicated by arrows at left. (b) A linear regression plot of log molecular mass (*y*‐axis) against relative migration distance (*x*‐axis) for each of the molecular mass standards and tested samples. Numbers and colors on the plots correspond to those in the gel image in panel a

Recent accumulating evidence from our own research suggests that homotetramer formation mediated by the N‐terminal domain of KinA is required for the kinase activity catalyzed by the C‐terminal domain, irrespective of nutrient availability (Eswaramoorthy et al., 2009, 2011). Conversely, most other studies argue that KinA forms a homo‐dimer as a functional unit (Dago et al., 2012; Lee et al., 2008; Szurmant & Hoch, 2013; Wang et al., 2001; Winnen et al., 2013). However, these reports used heterologous *E. coli* overexpression systems for preparation of a series of truncated KinA (e.g., C‐terminal domain, N‐terminal domain, and PAS‐A domain), which might not be physiologically relevant and therefore may not reflect the reactions that take place under physiological conditions in *B. subtilis*. To further provide solid supportive data to clarify the above discrepancies, we examined complex formation of KinA and its domain deletion mutants in a systematic manner in the native context of *B. subtilis* cells. First, *B. subtilis* cells harboring the IPTG‐inducible KinA‐GFP, KinA^ΔPAS‐A^‐GFP, KinA^ΔPAS‐AB^‐GFP, KinA^ΔPAS‐ABC^‐GFP (KinA_C_‐GFP), and KinA_N_‐GFP were cultured in an LB nutrient medium. To avoid heterocomplex formation between KinA expressed from the native locus and the GFP fusion protein from the IPTG‐inducible promoter, the native copy of the *kinA* gene was deleted from the host *B. subtilis* cells. To induce each of the GFP fusion proteins, IPTG was added to a mid‐logarithmic phase culture at a concentration of 10 μmol L^−1^, at which sporulation efficiency in the cells expressing the functional KinA‐GFP was similar to that in the starving wild‐type cells (i.e., not an IPTG‐inducible GFP fusion construct) as previously reported (Eswaramoorthy et al., 2009; Eswaramoorthy, Duan et al., 2010). The cell extracts were then incubated in the presence or absence of bis‐maleimidohexane (BMH), a protein cross‐linker specific for a free sulfhydryl group that was shown to be effective in detection of the wild‐type KinA tetramer (Eswaramoorthy et al., 2009). We note that KinA has five cysteine residues, one in PAS‐A, one in the boundary region between PAS‐A and PAS‐B, one in PAS‐B, and two in PAS‐C (Figure 1). In the absence of the cross‐linker, KinA and its truncated forms migrated as discrete bands with the molecular sizes expected for monomers (Figure 3a, odd numbered lanes). In the presence of the cross‐linker, new sets of larger products that migrated corresponding to the predicted tetramer sizes were detected in KinA‐GFP, KinA^ΔPAS‐A^‐GFP, and KinA_N_‐GFP (Figure 3a, lanes 2, 4, and 10). In the case of KinA^ΔPAS‐AB^‐GFP, however, the dimer size predominated in the presence of the cross‐linker and no clear band corresponding to the tetramer size was detected (Figure 3a, lane 6). No clear bands were detected for the C‐terminal autokinase domain (KinA^ΔPAS‐ABC^‐GFP) in the presence of the cross‐linker (Figure 3a, lane 8) since there are no cross‐linkable cysteine residues in the C‐terminal domain of KinA. Furthermore, results in Figure 3a, lanes 7 and 8 also indicated that two cysteine residues in GFP (GFPmut2) (Cormack, Valdivia, & Falkow, 1996) used as a tag are not involved in the complex formation. In Figure 3b, the relative migration distances and the expected molecular masses are plotted on the regression line.

**Figure 4:**
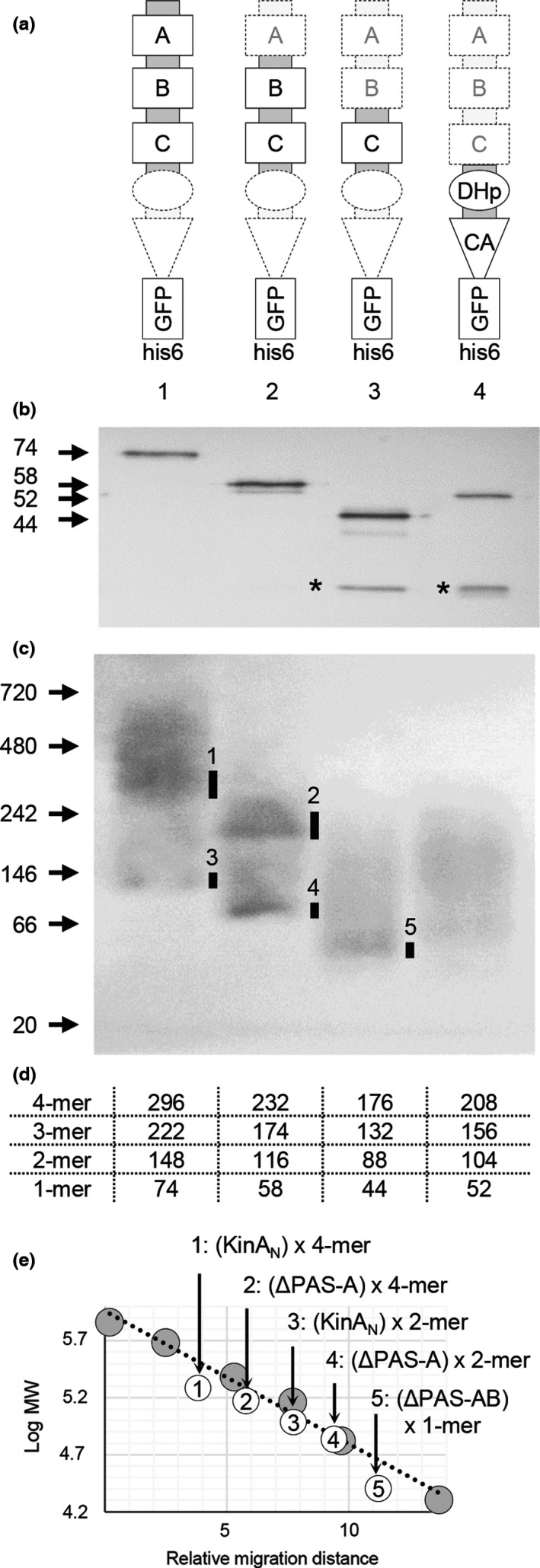
Complex formation of KinA and its truncated proteins analyzed by BN‐PAGE. (a) Proteins were overexpressed under the control of an IPTG‐inducible promoter in the KinA‐deleted (*ΔkinA*) *B. subtilis* strains MF7622 (KinA_N_‐GFP‐his6, 1), MF7623 (PAS‐BC‐GFP‐his6, 2), MF7624 (PAS‐C‐GFP‐his6, 3), and MF7625 (KinA_C_‐GFP‐his6, 4). (b) Overexpressed proteins were purified with Ni‐NTA beads and examined by 16% SDS‐PAGE to verify the size (arrows at left) and purity. An approximately 26‐kDa band corresponding to the GFP protein was detected besides the GFP fusion protein in lanes 3 and 4 (denoted as *). (c) Purified proteins were examined by BN‐PAGE (4–16% gradient gel). Size markers are indicated in kDa at left. Major bands are indicated with bars on the panel. (d) The molecular masses of the monomer (1‐mer), dimer (2‐mer), trimer (3‐mer), and tetramer (4‐mer) of the GFP‐his6‐tagged proteins are shown. (e) A linear regression plot of log molecular mass (*y*‐axis) against relative migration distance (*x*‐axis) for each of the molecular mass standards (gray circles) and tested proteins (open circles). Numbers in the open circles correspond to those in the gel image in panel C. Tested proteins are indicated as KinA_N_‐GFP‐his6 (N), PAS‐BC‐GFP‐his6 (ΔPAS‐A), PAS‐C‐GFP‐his6 (ΔPAS‐AB), and KinA_C_‐GFP‐his6 (C)

To verify our cross‐linking data, we performed BN‐PAGE analysis to separate native protein complexes on acrylamide gels based on size, taking advantage of the fact that the N‐terminal domain can form a tetramer (Eswaramoorthy et al., 2009). Thus, KinA_N_‐GFP‐his6 (N), PAS‐BC‐GFP‐his6 (ΔPAS‐A), PAS‐C‐GFP‐his6 (ΔPAS‐AB), and KinA_C_‐GFP‐his6 (C) were overexpressed from an IPTG‐inducible promoter in *B. subtilis* cells lacking the native *kinA* gene and purified using Ni‐NTA beads (Figure 4a). Each of the purified proteins was resolved on 16% SDS‐PAGE, followed by immunoblotting using anti‐GFP antibodies. The results verified that each of the purified proteins migrated with the molecular size expected for monomer under the denatured conditions (Figure 4b). On BN‐PAGE, two major bands were detected for KinA_N_ and PAS‐BC (Figure 4c, lanes 1 and 2). Based on these relative migration distances and the expected molecular masses plotted on the regression line with the known molecular masses of marker proteins, the upper and lower bands corresponded to tetramer and dimer, respectively (Figure 4c, d, and e, lanes 1 and 2). In contrast, PAS‐C migrated as a single band corresponding to the monomer size (Figure 4c, d, and e, lane 3). No discrete bands were detected for KinA_C_ (Figure 4c, d, and e, lane 4). We found that an approximately 26‐kDa band was detected besides the GFP fusion protein in lanes 3 and 4 (denoted as in Figure 4b). This protein might be a GFP protein, suggesting that some proteolytic enzymes may specifically cleave PAS‐C‐GFP‐his6 and KinA_C_‐GFP‐his6. The above results, together with the fact that KinA^ΔPAS‐AB^ is deficient in the autokinase activity (Eswaramoorthy et al., 2009; Eswaramoorthy, Duan et al., 2010), suggest that the homotetramer formed with KinA or KinA^ΔPAS‐A^ might be functional, but the dimer formed with KinA^ΔPAS‐AB^ might not. Finally, we note that the results suggest that the formation of these homotetramers is independent of autophosphorylation since the N‐terminal domain without autokinase domain is able to form a tetramer (Figure 3a, lane 10, and Figure 4).

### 3.3 Removal of the N‐terminal domain from KinA converts the autokinase into a phosphotransferase

**Figure 5:**
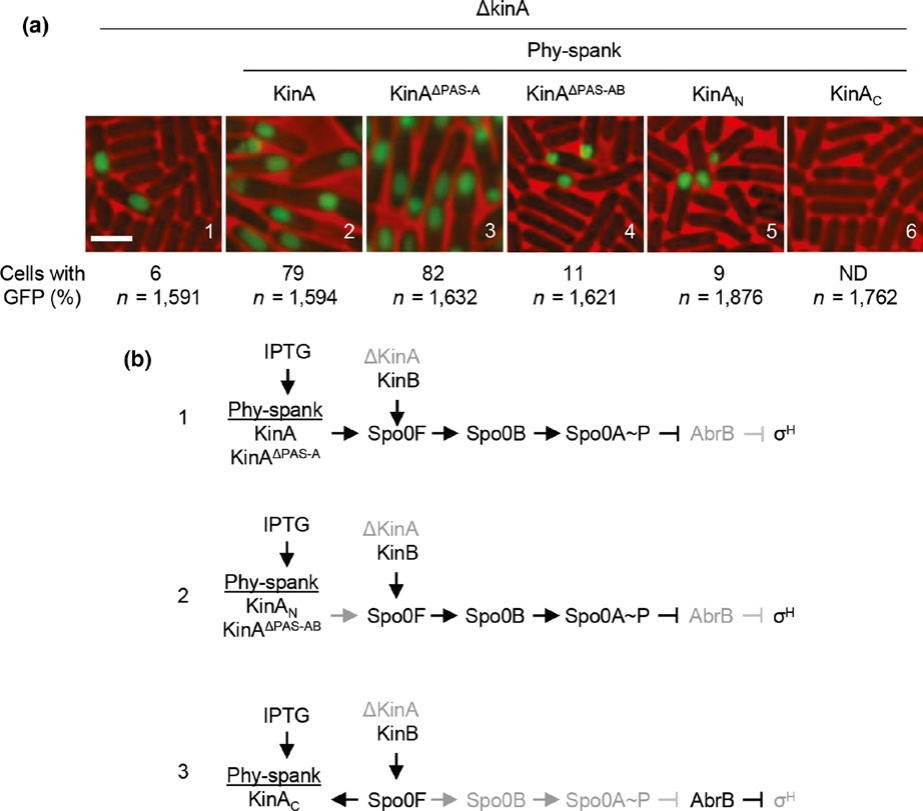
In vivo reverse phosphotransfer reaction assay. (a) Strains used for this assay were MF3386 (panel 1, control lacking the IPTG‐inducible construct), MF7679 (panel 2, IPTG‐inducible *kinA*), MF7680 (panel 3, IPTG‐inducible *kinA* ^*Δ*^ ^*PAS*^ ^*‐A*^), MF7681 (panel 4, IPTG‐inducible *kinA* ^*Δ*^ ^*PAS*^ ^*‐*^ ^*AB*^), MF3388 (panel 5, IPTG‐inducible *kin* *A*_*N*_), and MF3387 (panel 6, IPTG‐inducible *kin* *A*_*C*_). Each of the resulting strains has the gene for wild‐type KinB at the native locus and the IPTG‐inducible gene at the nonessential *amyE* locus on the chromosomal DNA in the *kinA*‐null background. A strain (MF3386) with the *kinA*‐null mutation and lacking the IPTG‐inducible construct was used as a control. A fluorescent sporulation reporter (*PspoIIQ‐gfp*) was introduced into the nonessential *thrC* locus on the chromosomal DNA of each tested strain. Cells were induced to sporulate by suspension in SM medium in the presence of 0.5 mmol L^−1^ IPTG for overexpression of the protein. Under this genetic system, KinB is expressed at physiological levels under the control of the native promoter, while KinA is not expressed in the *kinA*‐null background. Thus, sporulation can be induced by KinB to approximately 5–10% of the wild‐type level in the *kinA*‐null background. By adding IPTG, the cells overproducing the protein of interest can be observed to determine whether the level of sporulation is different from the control. The percentage of cells with the GFP signal and the total number of cells scored for GFP image analysis (n) are given. Bar, 2 μm. (b) The genetic systems for examination of the reverse phosphotransfer reaction. (Pathway 1) The overproduced functional KinA and KinA^Δ^ ^PAS^ ^‐A^ predominate over the native KinB, resulting in efficient sporulation (panels a2 and a3). (Pathway 2) The overproduced nonfunctional KinA_N_ and KinA^Δ^ ^PAS^ ^‐^ ^AB^ have no physical interaction with KinB. Thus, sporulation is induced via KinB (panels a4 and a5). (Pathway 3) The overproduced KinA_C_ is phosphorylated by transferring phosphoryl groups from Spo0F~P that are generated by KinB (panel A6)

It was demonstrated that the C‐terminal domain of KinA is phosphorylated by a reverse phosphotransfer reaction from the phosphorylated form of Spo0F under both in vitro (Wang et al., 2001) and in vivo conditions (Eswaramoorthy et al., 2009). On the basis of these notions and the data in the previous sections in this study, we hypothesized that the C‐terminal domain of KinA, as a monomer, accepts phosphoryl groups from Spo0F~P in a reverse phosphotransfer reaction. In other words, the N‐terminal domain, by forming a tetramer, might define the specificity of the enzyme activity. To test this hypothesis, we assayed sporulation efficiencies in the cells lacking the native KinA but overexpressing a series of truncated domains of KinA from an IPTG‐inducible promoter. It is generally known that, in the *kinA*‐null mutant (*ΔkinA*) background, sporulation efficiency decreases to approximately 5–10% of the wild‐type level under normal sporulation conditions. In this situation, KinB contributes to the phosphorelay by transferring phosphoryl groups to Spo0F. It is also noted that no physical interaction between KinA and KinB has been reported with the systematic yeast two‐hybrid screening using KinA or KinB as a bait (Fukushima, Yoshimura, Chibazakura, Sato, & Yoshikawa, 2006). Therefore, if the sporulation efficiency of cells expressing a certain domain is lower than that of the parental *ΔkinA* strain, it would suggest that the domain possesses reverse phosphotransfer activity (Eswaramoorthy et al., 2009). Using this “reverse phosphorylation” assay system, we systematically examined KinA, KinA^ΔPAS‐A^, KinA^ΔPAS‐AB^, KinA_N_, and KinA_C_ for reverse phosphorylation activity (Figure 5). To detect sporulation, we constructed a strain harboring a GFP reporter system for the forespore‐specific *spoIIQ* gene promoter controlled by σ^F^‐RNA polymerase. First, we verified that 6% of the parental *ΔkinA* cells showed GFP signals in the forespore compartment (Figure 5a, panel 1). When the wild‐type KinA (Figure 5a, panel 2) and KinA^ΔPAS‐A^ (Figure 5a, panel 3) were overexpressed by adding 0.5 mmol L^−1^ IPTG, the percentage of GFP‐positive cells increased, indicating that sporulation efficiencies significantly increased since these two proteins possess autokinase activity (Figure 5b, pathway 1). In contrast, when KinA^ΔPAS‐AB^ (Figure 5a, panel 4) and KinA_N_ (i.e., without C‐terminal domain, Figure 5a, panel 5) were overexpressed, sporulation efficiencies proved more or less similar to the parental *ΔkinA* strain (Figure 5a, panel 1). Finally, when KinA_C_ (i.e., without N‐terminal domain) was overexpressed (Figure 5a, panel 6), GFP signals were not detectable. These results indicate that neither KinA_N_ nor KinA^ΔPAS‐AB^ has any effect on the state of Spo0F phosphorylation, suggesting that the lack of physical interaction between KinA and KinB allows only for the forward transfer of phosphoryl groups toward Spo0A (Figure 5b, pathway 2). In contrast, there is evidence that the C‐terminal domain (KinAc) possesses phosphotransferase activity and acts upon KinB‐generated Spo0F~P, hindering the flow of phosphoryl groups through the relay. (Figure 5b, pathway 3) as reported (Eswaramoorthy et al., 2009). Taken together, these results suggest that removal of the N‐terminal domain from KinA converts the autokinase into a phosphotransferase as a result of the tetramer dissociation of the KinA_C_.

### 3.4 KinA activity is not regulated by the change of the NAD^+^/NADH ratio

A recent study by Kolodkin‐Gal et al., (2013) reports that, when KinA is purified under sporulation conditions, NAD^+^ binds to the PAS‐A domain of KinA. Based on these results and other assumptions, they propose that impaired oxidative phosphorylation leads to a drop in NAD^+^ levels, resulting in a conversion of the kinase from an inactive NAD^+^‐bound form to an active free form which can participate in biofilm formation (Kolodkin‐Gal et al., 2013). Another study reported by Gyan, Shiohira, Sato, Takeuchi, & Sato (2006) indicates that the NADH levels are relatively higher than those of NAD^+^ during growth and stationary phases. In general, NAD^+^ and NADH are ubiquitous and essential biomolecules, and thus these cellular concentrations are relatively high, ranging from 80 μmol L^−1^ (NADH) to 3 mmol L^‐1^ (NAD^+^) in *E. coli* (Bennett et al., 2009). We have previously reported that the KinA concentration is comparatively low (0.2–2 μmol L^−1^) during sporulation (Eswaramoorthy, Dinh, et al., 2010), while, to the best of our knowledge, the absolute cellular concentrations of the cofactors have not been reported in *B. subtilis*. Based on the available facts, we reasoned that, at any given moment during culture, the concentrations of NAD^+^ and NADH could be substantially higher than that of KinA. Under such conditions, if NAD^+^ specifically inhibits KinA with direct binding, there might be low‐affinity, but specific interactions between NAD^+^ and KinA.

**Figure 6:**
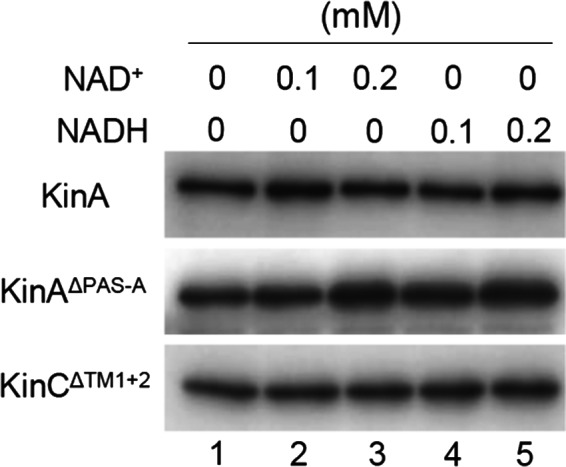
Effects of NAD ^+^ and NADH on the autophosphorylation of KinA, KinA^Δ^ ^PAS^ ^‐A^, and KinC^Δ^ ^TM^ ^1+2^. In vitro autophosphorylation assays were performed with each of the purified proteins (0.2 μmol L^−1^) in the presence of the indicated concentrations of NAD ^+^ or NADH. Reaction mixtures were analyzed by SDS‐PAGE followed by autoradiography

To clarify the above possibilities and provide direct evidence, we measured autokinase activities of the purified KinA and KinA^ΔPAS‐A^ from LB culture, in the presence of varying concentrations of NAD^+^ and NADH using an in vitro assay. As a control, KinC^ΔTM1+2^, the soluble and functional form KinC that is mainly involved in biofilm formation (Devi, Vishnoi, et al., 2015), was used. We found that autokinase activities of KinA and KinA^ΔPAS‐A^, as well as KinC^ΔTM1+2^, are constant in the presence of excess concentrations of NAD^+^ and NADH (up to 1000‐fold excess, similar to the in vivo conditions, Figure 6, lanes 3 and 5).

Finally, we directly determined the cellular concentrations of NAD^+^ and NADH during growth and sporulation (Table 1). To prevent undesired enzymatic degradation of NAD^+^ and NADH and also to recover the protein‐unbound free forms, the cell lysates were filtrated through a 3 kDa cutoff filter. In cells growing in rich medium (LB), the concentrations of NAD^+^ and NADH were approximately 3 mmol L^−1^ and 1 mmol L^−1^, respectively. In cells under sporulation conditions (DSM), the same conditions demonstrated by Kolodkin‐Gal, those concentrations were found to be approximately 1 mmol L^−1^ and 0.4 mmol L^−1^, respectively. As a control for the accuracy of this assay, we also measured the concentrations of the cofactors for *E. coli* cells grown in LB and DSM media. The assay returned values of approximately 2 mmol L^−1^ and 0.4 mmol L^−1^ for NAD^+^ and NADH, respectively, which are similar to those previously reported for *E*. *coli* under the same conditions (Bennett et al., 2009). These results indicate that NAD^+^ and NADH exist in approximately 1000‐fold excess of KinA (0.2–2 μmol L^−1^). Thus, these results suggest that the NAD^+^ and NADH ratio does not change significantly during growth and sporulation and, under such conditions, changes in the ratio between the two forms of the cofactors might not play a significant role in regulation of KinA activity, even if NAD^+^ does bind to the PAS‐A domain of the wild‐type KinA as reported (Kolodkin‐Gal et al., 2013).

**Table 1:** Cellular concentrations of NAD+ and NADH

Bacteria	Media	Concentration (mmol L^‐1^)		NAD^+^/NADH ratio
		NAD^+^	NADH	
*B. subtilis*	LB	3.04 ± 0.62	0.88 ± 0.48	3.5
	DSM	0.92 ± 0.38	0.38 ± 0.23	2.5
*E. coli*	LB	1.70 ± 0.14	0.41 ± 0.18	4.2
	DSM	1.23 ± 0.40	0.40 ± 0.16	3.1

## 4 Discussion

It has long been debated how the major sporulation kinase is activated upon starvation. A general model, like a simple ligand‐receptor model (Henry & Crosson, 2011) or a traditional bacterial two‐component system (Stock et al., 2000), has been proposed based on speculation that an as yet unidentified signaling molecule(s) produced only under starvation conditions binds directly to the N‐terminal “sensor” PAS domains, leading to the C‐terminus autokinase activity (Hoch, 1993, 2000; Stephenson & Hoch, 2002; Wang et al., 2001). However, to date, no ligand that directly interacts with the PAS domains and activates the autokinase activity has been reported. Alternatively, an as yet unidentified signaling molecule(s) produced only under nutrient‐rich conditions could bind directly to the N‐terminal PAS domains, interfering with the C‐terminus autokinase activity, in a similar fashion to bacterial chemotaxis (Hazelbauer, Falke, & Parkinson, 2008). Recently, it has also been suggested that NAD^+^ directly binds to the PAS‐A domain (Kolodkin‐Gal et al., 2013). While no direct supporting evidence is provided in that report, it is proposed that KinA activity is directly inhibited by the NAD^+^ binding to PAS‐A when the relative cellular concentration of the cofactors (NAD^+^/NADH) is high during growth in the presence of oxygen (Kolodkin‐Gal et al., 2013). Furthermore, the removal of the PAS‐A domain reportedly lowers the autokinase activity (Kolodkin‐Gal et al., 2013; Lee et al., 2008; Wang et al., 2001), contrary to the other studies (Eswaramoorthy & Fujita, 2010; Eswaramoorthy et al., 2009; Winnen et al., 2013). In support of the “sensor” model, the previous studies propose that PAS‐A acts as an important regulator of autokinase activity in response to environmental change (Kolodkin‐Gal et al., 2013; Lee et al., 2008; Wang et al., 2001).

In an attempt to conclude these debates, we have provided direct evidence in this study that (1) the N‐terminal domain alone is sufficient to form a tetramer without the C‐terminal autokinase domain, (2) among the three PAS domains (PAS‐ABC) in the N‐terminal domain, the PAS‐A domain is dispensable for tetramer formation, (3) the PAS‐C domain alone (i.e., in the absence of both PAS‐A and PAS‐B) is not able to form a tetramer, (4) the C‐terminal autokinase domain, without the N‐terminal domain, is not able to form a complex, and (5) NAD^+^ does not play a significant role in the regulation of KinA activity. These results reinforce the findings of previous studies that: (1) KinA forms a tetramer, mediated by the N‐terminal domain (Eswaramoorthy et al., 2009); (2) any two of the three PAS domains (PAS‐ABC) are sufficient to maintain the kinase activity (Eswaramoorthy & Fujita, 2010); (3) a chimeric kinase (YdaM_N_‐KinA_C_) composed of an N‐terminal domain of YdaM derived from *E. coli*, which is shown to be involved in tetramer complex formation (Lindenberg, Klauck, Pesavento, Klauck, & Hengge, 2013), fused to the C‐terminal domain of KinA, is able to phosphorylate Spo0A through the phosphorelay (Eswaramoorthy et al., 2011); (4) a small increase in the level of KinA beyond a certain threshold is able to trigger sporulation, regardless of culture conditions (Eswaramoorthy et al., 2011; Eswaramoorthy, Duan et al., 2010); and (5) autophosphorylation activity of KinA is independent of enzyme concentration (Eswaramoorthy et al., 2011). These results appear to be consistent with our hypothesis, in which the primary role of the N‐terminal domain of KinA is to form a tetramer as a functional unit, not to sense an unidentified starvation signal(s), and the threshold level of KinA acts as the trigger factor for entry into sporulation.

Our data in this study may rule out the possibility of NAD^+^ as an inhibitory ligand for KinA (Kolodkin‐Gal et al., 2013). It is known that NAD^+^ and NADH are involved in hundreds of reactions in cells, including not only central metabolic pathways (Holms, 1996) but also RNA metabolism and regulation (Jaschke, Hofer, Nubel, & Frindert, 2016). Thus, to maintain essential cellular functions, concentrations of the cofactors are generally high, within millimolar ranges (Bennett et al., 2009). In support of these notions, in growing and sporulating *B. subtilis*, cellular concentrations of free NAD^+^ and NADH (millimolar range) are approximately 1000‐fold higher than that of KinA (micromolar range as a monomer, but note that KinA is a tetramer) (Eswaramoorthy, Dinh, et al., 2010). Furthermore, the ratio between two cofactors’ concentrations does not significantly change between growth and sporulation conditions. In addition, our in vitro data indicate that the kinase activity is not inhibited in the presence of excess amounts of each of the cofactors. Therefore, the results in this study appear to be inconsistent with the previously proposed model, in which KinA is activated by a decrease in the NAD^+^/NADH ratio (Kolodkin‐Gal et al., 2013). While our data suggested that KinA is active regardless of the concentrations of the cofactors, in the future, detailed kinetic characterizations might be required to determine whether the previously proposed NAD^+^ binding to PAS‐A (Kolodkin‐Gal et al., 2013) is specific and physiologically relevant.

Recent studies using a combination of empirical and mathematical modeling suggest that cells’ growth rates decrease in response to nutrient starvation, leading to an increased cellular concentration of KinA and thus increased activation of Spo0A through the phosphorelay (Narula et al., 2016). In support of this model, it is important to note that the autophosphorylation activity of KinA is independent of enzyme concentration and also an as yet unidentified starvation signal(s), suggesting that the subunit assembly into a functional and constitutively active KinA tetramer is independent of the concentration of the individual protomers and the unknown starvation signal(s) (Eswaramoorthy et al., 2011). Thus, both empirical and modeling results suggest that the cellular concentration of the constitutively active KinA increases as its dilution slows down when cell growth ceases in response to nutrient starvation, thus, in turn, allowing the increase in KinA level to act as a trigger factor for phosphorelay activation. Furthermore, other studies suggest that Spo0A is activated in a pulsatile manner every cell cycle, resulting in a gradual increase in Spo0A activity over the course of multiple cell cycles (Levine, Fontes, Dworkin, & Elowitz, 2012). These pulsatile activations are controlled by the chromosomal arrangement of two phosphorelay genes, one (*spo0F*) located at the origin‐proximal part of the chromosome and the other (*kinA*) located near the terminus. During growth, replication of circular DNA starts at the origin, moves around the chromosome in both directions, and ends when the two replication forks meet each other on the opposite side of the chromosome. After replication starts but prior to cell division, a transient imbalance in gene dosage between origin‐proximal (*spo0F*) and terminus‐proximal (*kinA*) genes occurs, resulting in a temporary ratio of *spo0F*:*kinA* = 2:1. As a result, substrate inhibition of KinA by Spo0F occurs, leading to a delay in Spo0A activation through the phosphorelay (Narula et al., 2015).

Taken together, these recent findings suggest that the starvation event cannot be defined by the level of any single metabolite, but rather nutrients are consumed by cells, resulting in the production of a complex set of metabolites and the slowdown of cell growth. Thus, growth rate change in response to the available nutrients is a reasonable and accurate means of monitoring the availability of a variety of nutrients in the environment. Using this strategy, *B. subtilis* cells can integrate multiple sources of complicated environmental information into sporulation regulation by monitoring the cellular levels of KinA at the top of phosphorelay.

## Conflict of Interest

None declared.
