# Supplementary material for: The PAS domains of the major sporulation kinase in Bacillus subtilis play a role in tetramer formation that is essential for the autokinase activity
Source: Microbiologyopen. 2017 Apr 27;6(4):e00481. doi: 10.1002/mbo3.481 (PMC5552956; doi:10.1002/mbo3.481)
Supplement: Supplementary file 1 [file MBO3-6-na-s001.doc]

**Supplemental Material and Methods**

**The PAS domains of the major sporulation kinase in *Bacillus subtilis* play a role in tetramer formation that is essential for the autokinase activity**

Brittany Kiehler, Lindsey Haggett, and Masaya Fujita*

Department of Biology and Biochemistry, University of Houston, Houston, TX 77204-5001, U.S.A.

Running title: Role of PAS domain in the major sporulation kinase in *Bacillus subtilis*

*Address for correspondence: Masaya Fujita, Department of Biology and Biochemistry, University of Houston, Houston, TX 77204-5001, U.S.A., Phone: (713) 743-9479; e-mail: mfujita@uh.edu

Keywords: signal transduction, histidine kinase, response regulator, sporulation, *Bacillus subtilis*

**Supplemental Material and Methods includes**

Supplemental Materials and Methods

Supplemental References

Supplemental Tables S1-S3

**Strains**

The parent *B. subtilis* strain for all experiments was PY79. Details regarding the full genotypes of strains are provided in Table S1 in the supplemental material. Each of these mutations was transferred to competent cells of *B. subtilis* PY79. More detailed information is available upon request. The *E. coli* BL21(DE3) pET vector system (Novagen) was used for protein overexpression. *E. coli* DH5α was used for all DNA manipulations, including plasmid DNA constructions and preparations.

**Plasmid construction**

All plasmids were constructed in *E. coli* DH5α using basic molecular cloning methods and listed in Table S1 in the supplemental material.

**pMF679** was constructed by PCR amplifying the coding region of *kinA^ΔPAS-A^-his6* with primers op23 and om188 (corresponding to amino acid residues 136-606 with hexahistidine at the C-terminus) using chromosomal DNA of *B. subtilis* PY79 as a template. The PCR-amplified DNA was digested with *Hin*dIII and *Sph*I, and cloned into the same restriction sites of pDR111 . The resulting plasmid with flanking *amyE* sequences was integrated at the *amyE* locus in *B. subtilis* by transformation.

**pMF845** was constructed by PCR amplifying the coding region of *kinA_N_-gfp-his6* with primers op1 and om365 (corresponding to amino acid residues 1-394 with GFP followed by hexahistidine at the C-terminus) using chromosomal DNA of *B. subtilis* MF3360 as a template. The PCR-amplified DNA was digested *Hin*dIII and *Sph*I, and cloned into the same restriction sites of pDR111. The resulting plasmid with flanking *amyE* sequences was integrated at the *amyE* locus in *B. subtilis* by transformation.

**pMF846** was constructed by PCR amplifying the coding region of *PAS-BC-gfp-his6* with primers op23 and om365 (corresponding to amino acid residues 136-394 with GFP followed by hexahistidine at the C-terminus) using chromosomal DNA of *B. subtilis* MF3360 as a template. The PCR-amplified DNA was digested with *Hin*dIII and *Sph*I, and cloned into the same restriction sites of pDR111. The resulting plasmid with flanking *amyE* sequences was integrated at the *amyE* locus in *B. subtilis* by transformation.

**pMF847** was constructed by PCR amplifying the coding region of *PAS-C-gfp-his6* with primers op24 and om365 (corresponding to amino acid residues 259 to 394 with GFP followed by hexahistidine at the C-terminus) using chromosomal DNA of *B. subtilis* MF3360 as a template. The PCR-amplified DNA was digested with *Hin*dIII and *Sph*I, and cloned into the same restriction sites of pDR111. The resulting plasmid with flanking *amyE* sequences was integrated at the *amyE* locus in *B. subtilis* by transformation.

**pMF848** was constructed by PCR amplifying the coding region of *KinA_C_-his6* primers op3 and om365 (corresponding to amino acid residues 387 to 606 with GFP followed by hexahistidine at the C-terminus) using chromosomal DNA of *B. subtilis* MF3359 as a template. The PCR-amplified DNA was digested with *Hin*dIII and *Sph*I, and cloned into the same restriction sites of pDR111. The resulting plasmid with flanking *amyE* sequences was integrated at the *amyE* locus in *B. subtilis* by transformation.

**References**

Britton, R.A., P. Eichenberger, J.E. Gonzalez-Pastor, P. Fawcett, R. Monson, R. Losick & A.D. Grossman, (2002) Genome-wide analysis of the stationary-phase sigma factor (sigma-H) regulon of *Bacillus subtilis*. *J Bacteriol* **184**: 4881-4890.

Devi, S.N., B. Kiehler, L. Haggett & M. Fujita, (2015a) Evidence that Autophosphorylation of the Major Sporulation Kinase in *Bacillus subtilis* Is Able To Occur in trans. *J Bacteriol* **197**: 2675-2684.

Devi, S.N., M. Vishnoi, B. Kiehler, L. Haggett & M. Fujita, (2015b) In vivo functional characterization of the transmembrane histidine kinase KinC in Bacillus subtilis. *Microbiology* **161**: 1092-1104.

Eswaramoorthy, P., T. Guo & M. Fujita, (2009) In vivo domain-based functional analysis of the major sporulation sensor kinase, KinA, in *Bacillus subtilis*. *J Bacteriol* **191**: 5358-5368.

Fujita, M. & R. Losick, (2003) The master regulator for entry into sporulation in *Bacillus subtilis* becomes a cell-specific transcription factor after asymmetric division. *Genes Dev* **17**: 1166-1174.

Sambrook, J. & D.W. Russell, (2001) Molecular cloning : a laboratory manual, *In* Sambrook J, Russell, DW (ed), Cold Spring Harbor Laboratory, Cold Spring Harbor, N.Y.

Youngman, P., J.B. Perkins & R. Losick, (1984) Construction of a cloning site near one end of Tn917 into which foreign DNA may be inserted without affecting transposition in *Bacillus subtilis* or expression of the transposon-borne erm gene. *Plasmid* **12**: 1-9.

**Supplemental Tables**

| **Table S1. Strains used in this study.** | | |
| --- | --- | --- |
| **Parental strains** | | |
| Strain | Genotype or description | Reference |
| *B. subtilis* PY79 | prototroph wild type |  |
| *E. coli* DH5α | *fhuA2 lac(del)U169 phoA glnV44 Φ80' lacZ(del)M15 gyrA96 recA1 relA1 endA1 thi-1 hsdR17* |  |
|  |  |  |
| ***B subtilis* strains for crosslinking assay** | | |
| Strain | Genotype or description | Reference |
| MF3352 | *ΔkinA::tet ΔkinB::cm amyE::Phy-spank-kinA-gfp spc kan* |  |
| MF3353 | *ΔkinA::tet ΔkinB::cm amyE::Phy-spank-kinA^ΔPAS-A^-gfp spc kan* |  |
| MF3356 | *ΔkinA::tet ΔkinB::cm amyE::Phy-spank-kinA^ΔPAS-AB^-gfp spc kan* |  |
| MF3359 | *ΔkinA::tet ΔkinB::cm amyE::Phy-spank-kinA_C_ (^ΔPAS-ABC^)-gfp spc kan* |  |
| MF3360 | *ΔkinA::tet ΔkinB::cm amyE::Phy-spank-kinA_N_-gfp spc* |  |
|  |  |  |
| ***B subtilis* strains for Blue Native PAGE assay** | | |
| Strain | Genotype or description | Reference |
| MF7622 | *ΔkinA::tet amyE::Phy-spank-kinA_N_-gfp-his6 spc* | This study |
| MF7623 | *ΔkinA::tet amyE::Phy-spank-PAS-BC (ΔPAS-A)-gfp-his6 spc* | This study |
| MF7624 | *ΔkinA::tet amyE::Phy-spank-PAS-C (ΔPAS-AB)-gfp-his6 spc* | This study |
| MF7625 | *ΔkinA::tet amyE::Phy-spank-kinA_C_ (ΔPAS-ABC)-gfp-his6 spc* | This study |
|  |  |  |
| **Protein purification for in vitro assay** | | |
| Strain | Genotype or description | Reference |
| MF5268 | *ΔkinA::tet amyE::Phy-spank-kinA-his6 spc, B. subtilis* PY79 |  |
| MF5269 | *ΔkinA::tet amyE::Phy-spank-kinA^ΔPAS-A^-his6 spc, B. subtilis PY79* | This study |
| MF1226 | pGK10 *(PT7-spo0F-his6)* in *E. coli* BL21 (DE3) |  |
| MF1253 | pMF184 *(PT7-spo0B-his6)* in *E. coli* BL21 (DE3) |  |
| MF2488 | pMF338 *(PT7-spo0A-his6)* in *E. coli B*L21 (DE3) |  |
| MF4516 | pMF599 *(PT7-KinC^ΔTM1+2^)* in *E. coli* BL21 (DE3) |  |
|  |  |  |
| ***B subtilis* strains for reverse phosphotransfer assay** | | |
| Strain | Genotype or description | Reference |
| MF3386 | *ΔkinA::tet thrC::PspoIIQ-gfp erm* | This study |
| MF3388 | *ΔkinA::tet amyE::Phy-spank-kinA_N_ spc thrC::PspoIIQ-gfp erm* | This study |
| MF3387 | *ΔkinA::tet amyE::Phy-spank-kinA_C_ spc thrC::PspoIIQ-gfp erm* | This study |
| MF7679 | *ΔkinA::tet amyE::Phy-spank-kinA spc thrC::PspoIIQ-gfp erm* | This study |
| MF7680 | *ΔkinA::tet amyE::Phy-spank-kinA^ΔPAS-A^ spc thrC::PspoIIQ-gfp erm* | This study |
| MF7681 | *ΔkinA::tet amyE::Phy-spank-kinA^ΔPAS-AB^ spc thrC::PspoIIQ-gfp erm* | This study |

| **Table S2. Plasmids used in this study** | | |
| --- | --- | --- |
| Plasmid | Description | References |
| pMF416 | *amyE::Phy-spank-kinA spc* |  |
| pMF362 | *amyE::Phy-spank-kinA_C_ spc* |  |
| pMF446 | *amyE::Phy-spank-kinA_N_ spc* |  |
| pMF369 | *amyE::Phy-spank-kinA^ΔPAS-A^ spc* |  |
| pMF433 | *amyE::Phy-spank-kinA^ΔPAS-AB^ spc* |  |
| pMF679 | *amyE::Phy-spank-kinA^ΔPAS-A^-his6* | This study |
| pMF845 | amyE::Phy-spank-*kinA_N_-gfp-his6* | This study |
| pMF846 | *amyE::Phy-spank-PAS-BC-gfp-his6* | This study |
| pMF847 | *amyE::Phy-spank-PAS-C-gfp-his6* | This study |
| pMF848 | *amyE::Phy-spank-kinA_C_-gfp-his6* | This study |

| **Table S3. Oligonucleotide primers used in this study** | |  |
| --- | --- | --- |
| Primer | Sequence | References |
| op1 | 5’-gccaagcttacataaggaggaactactatggaacaggatacgcagcatgtt-3’ |  |
| op3 | 5'-gccaagcttacataaggaggaactactatgctgaaatcggaaaaattatca-3' |  |
| op23 | 5’-gccaagcttacataaggaggaactactatggaatcgactacatatataacggat-3’ |  |
| op24 | 5’-gccaagcttacataaggaggaactactatgcaaaccatcctgcaaaaaagccgt-3’ |  |
| om188 | 5'-cgggcatgcttagtggtggtggtggtggtgtttttttggaaatgaaattttaaacgc-3' |  |
| om365 | 5’-gcggcatgcttagtggtggtggtggtggtgtttgtatagttcatccatgcc-3’ | This study |
